# Brote de *tinea capitis* y otras micosis superficiales en una comunidad urbana de Medellín

**DOI:** 10.7705/biomedica.6900

**Published:** 2023-08-31

**Authors:** Nelson Andrés Sterling, Dubán Andrés Rincón, Sebastián Barrera, Erika Andrea Sánchez, Diana Yuledi Molina, Martha Eugenia Urán, María del Pilar Jiménez

**Affiliations:** 1 Facultad de Medicina, Universidad de Antioquia, Medellín, Colombia Universidad de Antioquia Facultad de Medicina Universidad de Antioquia Medellín Colombia; 2 Grupo Micología Médica, Departamento de Microbiología y Parasitología, Facultad de Medicina, Universidad de Antioquia, Medellín, Colombia Universidad de Antioquia Grupo Micología Médica, Departamento de Microbiología y Parasitología Facultad de Medicina Universidad de Antioquia Medellín Colombia

**Keywords:** dermatomicosis, tiña, tiña del cuero cabelludo, brotes de enfermedades, Dermatomycoses, tinea, tinea capitis, disease outbreaks

## Abstract

**Introducción.:**

Las dermatofitosis son infecciones fúngicas superficiales de epitelios queratinizados. La *tinea capitis* es una de ellas y afecta a poblaciones escolares vulnerables. Carpinelo es un barrio del área periférica de Medellín con precarias condiciones socioeconómicas. Ante la sospecha de un brote de dermatofitosis, los afectados fueron evaluados.

**Objetivo.:**

Evaluar clínica y microbiológicamente pacientes del barrio Carpinelo con sospecha de micosis cutáneas para determinar la presencia de un brote por dermatofitos.

**Materiales y métodos.:**

Se llevó a cabo un estudio descriptivo, de corte longitudinal, con muestreo a conveniencia. Se hizo una búsqueda activa de casos en el Jardín Educativo Buen Comienzo de Carpinelo en niños de la institución y sus familiares. Se evaluaron clínicamente y se tomaron muestras de escamas y cabellos para exámenes directos y cultivos microbiológicos. Se analizó el perfil demográfico, clínico y micológico, con el programa estadístico SPSS™, versión 25.

**Resultados.:**

Se estudiaron 57 pacientes, 47 eran menores de edad con una media de edad de seis años; se observó una proporción de hombres y mujeres de 2:1. Los pacientes con resultados positivos se diagnosticaron con *tinea capitis* (78,95 %), *tinea faciei* (15,79 %) y *tinea corporis* (10,52 %). El 75,43 % de los pacientes recibió tratamiento previo y de estos el 69,73 % fue con esteroides. El examen directo fue positivo en el 53,84 % y los cultivos en el 46,5 % de los casos. Los agentes aislados fueron: *Microsporum canis* (77,77 %), *Trichophyton* spp. (11,11 %), *Trichophyton rubrum* (5,55 %) y *Malassezia* spp. (5,55 %).

**Conclusión.:**

*Tinea capitis* fue la presentación clínica más común y *M. canis e*l dermatofito más frecuentemente aislado. Llamó la atención el uso de esteroides como primera y única opción del tratamiento empírico, lo cual resalta la importancia del diagnóstico microbiológico para proporcionar la terapia apropiada.

## Abstract (en)

**Introduction.:**

Dermatophytoses are superficial fungal infections of the keratinized epithelium like *tinea capitis.* The latte mainly affects school-vulnerable populations. Carpinelo is a peripheral neighborhood in Medellín with poor socioeconomic conditions and where a suspected *tinea capitis* outbreak took place.

**Objective.:**

To study and characterize, clinically and microbiologically, patients with suspected dermatophytosis in Carpinelo.

**Material and methods.:**

We carried out a descriptive and longitudinal study with an active case search of *tinea capitis* in children and their relatives from the *Jardín Educativo Buen Comienzo* community in Carpinelo. Patients were clinically evaluated, and samples of scales and hair were taken to perform mycological studies with a 10 % potassium hydroxide and culture in Sabouraud and Mycosel agar. We analyzed the data with the statistical program SPSS™. 25 version.

**Results.:**

Fifty-seven individuals were studied: 47 were children with a mean age of six years and a ratio of 2:1 male to female. Patients with confirmed diagnosis presented the following clinical forms: *tinea capitis* (78.95%), *tinea faciei* (15.79%) or *tinea corporis* (10.52%). Out of the total, 69.76% of the patients had previous treatment with steroids. The direct test was positive in 53.84% of the samples, and 46.15% had positive cultures. The isolated species were: *Microsporum canis* (77.77%), *Trichophyton* spp. (11.11%), *Trichophyton rubrum* (5.55%), and *Malassezia* spp. (5.55 %).

**Conclusion.:**

*Tinea capitis* was the most common clinical form, and *M. canis* was the most frequently isolated species. The use of steroids as the first and only option for empiric treatment was worth of notice. The findings of this study point out the importance of microbiological diagnosis in choosing the best treatment for the patients.

Los dermatofitos son hongos filamentosos, hialinos, tabicados, queratinofílicos, con capacidad de invadir tejidos queratinizados derivados de la epidermis y generar infecciones en la piel, el cabello y las uñas. Son causantes de algunas de las enfermedades infecciosas más frecuentes en el hombre ^1^ con una amplia gama de cuadros clínicos según su lugar de aparición, y con un espectro clínico que va desde manifestaciones leves hasta lesiones inflamatorias ^1^^,^^2^.

Las infecciones producidas por este grupo de hongos son generalmente superficiales y afectan principalmente el estrato córneo de la piel ^3^. Estos hongos se pueden clasificar en función de sus hábitats naturales como antropofílicos, zoofílicos y geofílicos, y su origen se condiciona según la epidemiología y la clínica de las lesiones que producen ^3^.

Entre los factores de riesgo descritos para estas infecciones encontramos el contacto con animales o personas infectadas, o con fómites, como se ha descrito en brotes estudiados en centros de cuidado infantil y en escuelas ^4^^,^^5^.

En este estudio se describen las características clínicas y microbiológicas de un brote de micosis superficiales en una comunidad con condiciones socioeconómicas bajas en el barrio Carpinelo de Medellín entre septiembre y diciembre del 2018.

## Materiales y métodos

Se llevó a cabo un estudio descriptivo, de corte longitudinal, con un muestreo a conveniencia. Se adelantó una búsqueda activa de casos en el Jardín Educativo Buen Comienzo de la Alcaldía de Medellín del barrio Carpinelo, comuna uno de Medellín. Se evaluaron 57 pacientes. Los 57 pacientes estaban conformados por 47 menores de edad, 8 adultos que trabajaban en el jardín infantil -dos profesoras, un auxiliar de cuidado, una persona de servicios generales, una persona en oficios varios, un constructor y dos madres de los niños del jardín infantil- y 2 mascotas cuyos propietarios eran familias de los niños que asistían al jardín infantil.

Se realizaron dos jornadas de salud en dos días diferentes, llevados a cabo por estudiantes de la Facultad de Medicina, médicos, profesores y personal adscrito al Departamento de Microbiología y Parasitología de la Facultad de Medicina de la Universidad de Antioquia.

A los pacientes con sospechas de micosis cutáneas se les explicó en qué consistía el procedimiento del examen clínico y la toma de muestras para la evaluación microbiológica.

Los adultos responsables de los menores de edad diligenciaron y firmaron el consentimiento informado, aprobado para este fin, por el Comité de Ética de la Facultad de Medicina de la Universidad de Antioquia. La recolección de los datos demográficos y epidemiológicos se realizó mediante el diligenciamiento de formularios estructurados.

Para la evaluación médica durante la brigada se seleccionaron los individuos con lesiones características de las micosis superficiales en cualquier parte del cuerpo, que tuviesen forma de placas descamativas con borde eritematoso, con vesículas o sin ellas, con costras o sin ellas, con centro limpio o sin él, y con máculas o manchas hipocrómicas o hipercrómicas; en el cuero cabelludo se buscaron lesiones en forma de placa, descamativas, alopécicas o no, con bordes eritematosos, con costras o sin ellas y con foliculitis o sin ella. Después de hacer esta selección se encontró que 57 pacientes presentaban una o varias de estas lesiones.

Se procedió a la evaluación clínica y al diligenciamiento de los formatos que permitían recolectar la información sociodemográfica, la historia clínica, los antecedentes personales y los tratamientos previos. Luego, se procedió a la toma de muestras de escamas mediante raspado con bisturí número 15 o 20 y a la obtención de cabellos de las lesiones de piel y anexos.

Para practicar el examen directo, las muestras se procesaron con hidróxido de potasio al 10 % y se tiñeron con azul de Evans o tinción de Gram. Se observaron al microscopio y se hizo la descripción morfológica de las estructuras fúngicas y bacterianas en los casos en los que se visualizaron.

Se sembraron muestras pareadas para cultivo en agar de Sabouraud y Mycosel™, excepto en los casos con sospecha de pitiriasis versicolor, cuando las muestras se sembraron en agar Dixon. Los medios sembrados se incubaron a temperatura ambiente durante 30 días y se analizaron periódicamente cada ocho días. En caso de no observar crecimiento en este tiempo, las muestras se cultivaron por 20 o 30 días más, solo en los casos en los que persistía sospecha de infección por dermatofito dado los resultados positivos del examen físico y el examen directo; las otras muestras se consideraron negativas a los 30 días.

Para la confirmación del agente etiológico, se realizó examen directo a las colonias, previa tinción con azul de lactofenol. A las muestras con crecimiento sugestivo de dermatofitos y sin esporulación, se les realizó una nueva siembra en agar arroz y agar papa-dextrosa. Se incubaron durante 10 días (o 20 días en algunos casos) a temperatura ambiente. Posterior a esto, se realizó una nueva placa con azul de lactofenol para observar las estructuras que permitieran la confirmación del respectivo género y especie.

En los casos en los que se observó crecimiento de colonias sugestivas de *Trichophyton* spp. en los medios de Mycosel™ y Sabouraud, se realizó la caracterización de la especie de dermatofito mediante su cultivo en agar lactosa púrpura de bromocresol, agar papa-dextrosa, urea y agar T1 (medio basal sin vitaminas) y T4 (medio con tiamina) durante ocho días.

El análisis estadístico descriptivo se hizo utilizando el programa IBM SPSS Statistics™, versión 25. Se utilizó la prueba de Kolmogorov-Smirnov para determinar la normalidad de las variables cuantitativas. Se comparó el valor de las medianas mediante la prueba de Mann-Whitney cuando las variables no tuvieron una distribución normal. Las asociaciones entre las variables cualitativas se analizaron mediante la prueba de ji al cuadrado (c^2^). El nivel de significancia estadística adoptado fue del 5 % (p<0,05).

## Resultados

Se estudiaron 57 pacientes de la localidad de Carpinelo con sospecha clínica de dermatofitosis: 47 menores de edad, 8 adultos y 2 mascotas. Se tomaron 118 muestras, entre escamas (88) y cabellos (30), de 88 lesiones. De los 57 pacientes evaluados, 38 tuvieron resultados negativos en los exámenes de laboratorio. Los exámenes directos y los cultivos de las dos mascotas fueron negativos a pesar de presentar lesiones clínicamente compatibles con un proceso infeccioso de etiología fúngica. Sólo en dos de los ocho pacientes adultos, con lesiones sugestivas de micosis superficiales, se logró confirmar el diagnóstico por resultados de laboratorio, mientras que 30 de los 47 menores tuvieron resultados negativos en los exámenes directos de los cultivos de escamas y de cabellos. En resumen, los resultados de los exámenes de laboratorio permitieron confirmar la etiología fúngica de las micosis superficiales en 19 de los 57 pacientes, con un total de 39 muestras positivas.

Los rangos de edad en los 47 menores de edad evaluados oscilaron entre los 7 meses y los 16 años, con una media de edad de 6 años y una proporción entre hombres y mujeres de 2:1 (cuadro 1).

**Cuadro 1:** Distribución por sexo y edad de los pacientes atendidos en la brigada de salud en el jardín infantil Buen Comienzo de la localidad de Carpinelo, Medellín, septiembre del 2018

Edad (años)	Femenino (n)	Masculino(n)	Total [n (%)]
Menores de edad			
<2	0	1	1 (1,75)
2-5	11	17	28 (49,12)
6-11	6	8	14 (24,56)
12-16	0	4	4 (7,02)
Total	17	30	47 (82,45)
Adultos			
22	1	0	1 (1,75)
25	1	0	1 (1,75)
30	1	0	1 (1,75)
33	1	0	1 (1,75)
34	1	0	1 (1,75)
39	1	0	1 (1,75)
42	1	0	1 (1,75)
58	0	1	1 (1,75)
Total	7	1	8 (14,04)
Mascotas			
<2	0	1	1 (1,75)
2-5	0	0	0
6-11	1	0	1 (1,75)
Total	1	1	2 (3,51)

**Figura 1:**
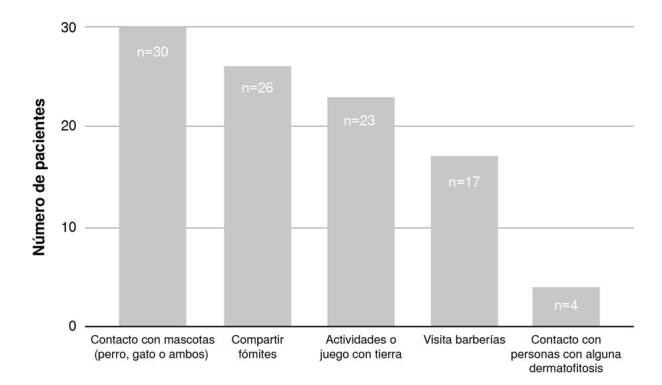
Factores de riesgo en los pacientes evaluados

Se indagó con los pacientes o con los adultos responsables del cuidado de los menores, por la posible exposición a factores de riesgo como contacto con mascotas (perros, gatos o ambos), fómites, actividades o juego con tierra, visitas a barberías o contacto con personas que presentaran lesiones compatibles con una dermatofitosis. Se encontró que el 80,71 % (n=47) de los pacientes con sospecha clínica había estado expuesto a algún factor de riesgo, mientras que el 42,11 % (n=24) refirió haber estado expuesto a tres o más factores de riesgo (figura 1).

Cuando se les preguntó a los pacientes por tratamientos previos durante la evolución de la enfermedad, el 75,43 % (n=43) manifestó haber usado algún tratamiento previo a la evaluación clínica y a la toma de la muestra (cuadro 2). El uso de esteroides fue el tratamiento más frecuentemente usado por el 69,76 % (n=30) de los pacientes: solos en el 37,2 % (n=16) o en combinación con antimicóticos y antibióticos en el 32,55 % (n=14) (cuadro 3). De los pacientes con diagnóstico confirmado, el 94,73 % (n=18) refirió tratamiento previo con antimicóticos, mientras que el 77,77 % (n=14) se había tratado previamente con esteroides solos o en combinación. En cuanto al uso de antimicóticos, la mayoría de los pacientes reportó su uso tópico: solo en el 13,95 % (n=6) o en combinación con esteroides y/antibióticos en el 32,55 % (n=14).

**Cuadro 2:** Distribución de los pacientes según la exposición a tratamientos previos a la evaluación clínica y a la toma de las muestras para los exámenes de laboratorio

	Confirmación diagnóstica de micosis superficiales		
Tratamiento previo	Negativo n (%)	Positivo n (%)	Total n (%)
Si	25 (43,85)	18 (31,57)	43 (75,43)
No	2 (3,50)	1 (1,75)	3 (5,26)
Sin información	11 (19,29)	0	11 (19,29)
**Total**	38 (66,66)	19 (33,33)	57 (100)

**Cuadro 3:** Tratamientos previos a la evaluación clínica y microbiológica

	Confirmación de diagnóstico de micosis cutánea		
Tratamiento previo	Negativo n	Positivo n	Total n
Esteroides	8	8	16
Antimicóticos	3	3	6
Antibióticos	2	0	2
Esteroides más antimicóticos	6	3	9
Esteroides más antimicóticos más antibióticos	2	3	5
Tratamientos caseros	4	0	4
Queratinolíticos	1	0	1
Total	26	17	43

* Tratamientos caseros: vinagre, vaselina, hipoclorito de sodio diluido o ajo

De los pacientes con resultados positivos en los exámenes de laboratorio, el 16,66 % (n=3) usó antimicóticos solos y el 33,33 % (n=6) los usó en combinación con esteroides o antibióticos. Nueve (50 %) pacientes con diagnóstico confirmado usó antimicóticos antes de la evaluación clínica y microbiológica (cuadro 3). Sin embargo, tres pacientes con diagnóstico confirmado de querion de Celso, *tinea capitis* o pitiriasis versicolor, tomaron fluconazol oral por periodos mayores a un mes y medio o más (antes de la evaluación clínica y microbiológica), pero no presentaron mejoría de sus cuadros clínicos.

**Figura 2:**
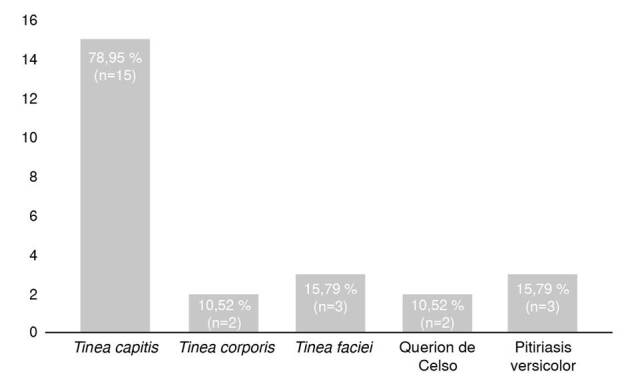
Formas clínicas presentadas por los pacientes en quienes se confirmó el diagnóstico de micosis cutáneas por exámenes de laboratorio.

De las presentaciones clínicas encontradas en los pacientes con diagnóstico de micosis confirmado, la *tinea capitis* fue la más común en el 78,95 % (n=15) de los casos, dos pacientes presentaron querion de Celso (10,52 %), tres presentaron *tinea faciei* (15,79 %) y dos tenían *tinea corporis* (10,52 %). En dos (10,52 %) pacientes se confirmó el diagnóstico de pitiriasis versicolor (figura 2).

**Figura 3:**
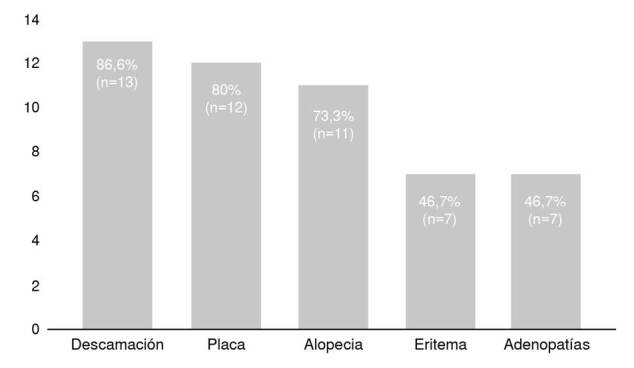
Características de las lesiones de los pacientes en quienes se confirmó microbiológicamente el diagnóstico de *tinea capitis*

En el examen físico se hallaron adenomegalias en el 35,9 % (n=14) de los pacientes, localizadas principalmente en la región cervical. Las características más comunes de las lesiones, en las diferentes formas clínicas, fueron descamación y eritema. En la figura 3 se presentan las características de las lesiones en los pacientes con diagnóstico confirmado de *tinea capitis*.

En el examen directo con hidróxido de potasio al 10 % y azul de Evans, se observaron estructuras fúngicas (hifas hialinas septadas o tabicadas artroconidias) en el 36,84 % (n=7), invasión de cabellos en el 57,89 % (n=11) y blastoconidias unigemantes en el 10,52 % (n=2). De las 18 muestras con crecimiento de cultivo se aisló *M. canis* en el 77,77 % (n=14), *T. rubrum* en el 5,55 % (n=1), *Trichophyton* spp. en el 11,11 % (n=2) y *Malassezia* spp. en el 5,55 % (n=1).

En los hallazgos de los exámenes directos relacionados con el agente aislado en cultivo de las muestras de los pacientes en quienes se confirmó el diagnóstico por el laboratorio, se observó lo siguiente: en las muestras de seis pacientes al examen directo se observó invasión endotrix-ectotrix y de estas muestras se aisló en cultivo *M. canis*. En las muestras de otros dos pacientes se observaron en el examen directo hifas hialinas septadas o tabicadas, y de ellas se obtuvo a *M. canis* en cultivo; en las muestras de otros tres pacientes de las cuales creció *M. canis*, se observaron artroconidias en el examen directo; en un paciente, a pesar de que los resultados de los exámenes directos fueron negativos, se pudo aislar *M.canis* en cultivo.

Para las muestras de diez pacientes, solo después de ocho semanas en cultivo, se pudo identificar el agente causal: *M. canis* (n=7) y *Trichophyton* spp. (n=3).

## Discusión

Las infecciones fúngicas de piel y anexos constituyen uno de los grupos más numerosos y diversos de todas las micosis. La prevalencia de las micosis superficiales ha aumentado en las últimas décadas (20-25 % de la población mundial) y se ha convertido en una de las infecciones más frecuentes en el humano ^6^^,^^7^. Sus agentes causales, los dermatofitos, sobreviven a temperaturas entre los 25 y los 28 °C, por esta razón, países tropicales como Colombia son adecuados para su supervivencia ^6^^,^^7^. La frecuencia y distribución de las formas clínicas producidas por los dermatofitos varían de acuerdo con la ubicación geográfica y los factores socioeconómicos, culturales y migratorios ^8^. Estas enfermedades pueden adquirirse por contacto con humanos infectados (antropofílicas), animales infectados (zoofílicas) y por contacto con la tierra (geofílicas); y se clasifican según su localización en el cuerpo ^9^.

Es así como en este reporte observacional, llevado a cabo en la localidad de Carpinelo de Medellín, se evaluaron 57 pacientes con sospecha clínica de dermatofitosis. De ellos, 47 pacientes eran menores de edad con un rango entre los 7 meses y los 16 años, y una media de edad de 6 años. La forma clínica más común de las dermatofitosis en este grupo fue la *tinea capitis* (78,95 %), seguida de la *tinea faciei* en 15,79 % y la *tinea corporis* (10,52 %).

Estos datos contrastan con lo encontrado en una investigación con estudiantes de nivel primario en Ayacucho (Perú), en el cual se encontró una frecuencia de 68 % de dermatofitosis en la población estudiada, con la siguiente distribución anatómica de las formas clínicas: 47 %, *tinea pedis* (espacios interdigitales de los pies); 29,4 %, *tinea faciei*; 19,1 %, *tinea corporis* y 4,4 %, *tinea capitis*^10^. En un estudio realizado en Colombia, por Alvarado y colaboradores, en un periodo de 13 años (2000 a 2012), en pacientes con edades entre los 0 y los 18 años, encontraron una frecuencia de *tinea unguium* de 29,6 % y de *tinea capitis* de 28,4 % ^11^.

*Microsporum canis* fue el principal agente etiológico aislado en la población de este estudio, lo cual contrasta con observaciones realizadas en otros. En una investigación realizada en Etiopia, en estudiantes de escuela básica primaria, el agente etiológico predominante fue *Trichophyton violaceum*^12^, mientras que en pacientes pediátricos de la India, *T. mentagrophytes* fue la especie identificada con mayor frecuencia en todos los sitios, excepto en el cuero cabelludo, donde fue más común la identificación de *T. tonsurans*^13^. En los últimos 10 a 20 años, *T. tonsurans* ha sido el microogranismo más frecuentemente aislado en los casos de *tinea capitis* en Norteamérica y Centroamérica, y en algunas regiones de Europa y África ^14^. En Bélgica se llevó a cabo un estudio reciente para evaluar los agentes causales de *tinea capitis* y los autores encontraron que los dermatofitos antropofílicos *M. audouinii* y *T. soudanense* eran los más frecuentes ^15^. En niños de República Dominicana con *tinea capitis* se aisló *T. tonsurans* en el 61,16 % de los casos, *M. audouinii* en el 24,27 % y *M. canis* en el 11,65 % de los pacientes ^16^.

En Colombia, en el estudio mencionado, realizado por Alvarado y Pereira en Bogotá, se evidenció que *T. rubrum* fue el dermatofito más aislado en los casos de *tinea unguium*, seguido de *M. canis* en los casos de *tinea capitis* (89,7 %) ^11^. Los datos de estos reportes y los del presente trabajo confirman la variabilidad del agente causal según la región geográfica.

En esta investigación, la forma clínica predominante fue la *tinea capitis* y el agente causal más aislado fue *M. canis,* lo que coincide con hallazgos de otro reporte de Medellín en el que *M. canis* fue hallado en el 86 % de los pacientes evaluados con esta condición ^17^.

Algunos de los factores de riesgo más frecuentes en la población estudiada fueron el contacto con mascotas -perro, gato o ambos- (30 %), el contacto con fómites (26 %) y las actividades o juegos con tierra (23 %). La alta frecuencia de contacto con mascotas como factor de riesgo contrasta con lo encontrado en un estudio en Camerún, en población infantil con *tinea capitis*, en el cual la presencia de animales no mostró tener una relevancia estadística en el desarrollo de esta infección ^18^. Por el contrario, la baja frecuencia de contacto con personas afectadas por dermatofitosis se diferencia de lo encontrado en estudiantes de área rural de Eskisehir (Turquía) donde se evidenció que la presencia de dermatofitosis en la familia era un factor de riesgo para el desarrollo de esta en los estudiantes ^19^, hallazgo también descrito en el estudio realizado en Bélgica ^15^.

En los resultados presentados en este reporte se destaca el porcentaje de pacientes que había usado o estaba usando esteroides, problema que ya ha sido tratado por otros autores, quienes evidencian que el uso frecuente de estos, de forma empírica, trae como consecuencia formas atípicas de las micosis, de difícil diagnóstico y que llevan a la cronicidad de las lesiones ^20^^,^^21^, persistencia y recurrencia de los cuadros clínicos ^22^^,^^23^, así como una mayor penetración del hongo en la dermis ^20^^,^^21^ y con esta secuelas irreversibles.

En resumen, en este estudio la *tinea capitis* fue la presentación clínica más común y *M. canis* el agente causal más frecuente. Llamó la atención el uso de esteroides como primera y única opción de tratamiento empírico, lo que resalta la importancia del diagnóstico microbiológico para proporcionar la terapia apropiada.

Es importante señalar que el número de casos de micosis superficiales que se confirmaron clínica y microbiológicamente en este estudio, se hizo en una ventana de tiempo muy corta y restringida a un solo lugar. Por esta razón, se planteó a la Secretaría de Salud del municipio de Medellín, la importancia de hacer búsqueda y vigilancia activa de estas infecciones en los otros jardines educativos de la Alcaldía de Medellín.
